# Charlson Comorbidity Index is correlated with all-cause readmission within six months in patients with heart failure: a retrospective cohort study in China

**DOI:** 10.1186/s12872-023-03151-9

**Published:** 2023-03-06

**Authors:** Song Sheng, Feng-qin Xu, Yan-hong Zhang, Ye Huang

**Affiliations:** 1 Emergency Department, Xiyuan Hospital, China Academy of Chinese Medical Sciences, Beijing, 100091 China; 2 Institute of Geriatrics, Xiyuan Hospital, China Academy of Chinese Medical Sciences, Beijing, 100091 China

**Keywords:** Charlson Comorbidity Index, Heart failure, All-cause readmission within six months, Threshold effect, Retrospective cohort study

## Abstract

**Background:**

Charlson Comorbidity Index (CCI) is positively associated with all-cause readmission in patients with heart failure (HF) in western countries. However, there is a scarcity of strong scientific evidence supporting the correlation in China. This study aimed at testing this hypothesis in Chinese.

**Methods:**

We conducted a secondary analysis of 1,946 patients with HF in Zigong Fourth People’s Hospital in China between December 2016 to June 2019. Logistic regression models were used to study the hypotheses, with adjustments for the four regression models. We also explore the linear trend and possible nonlinear relationship between CCI and readmission within six months. We further conducted subgroup analysis and tests for interaction to examine the possible interaction between CCI and the endpoint. Additionally, CCI alone and several combinations of variables based on CCI were used to predict the endpoint. Under the curve (AUC), sensitivity and specificity were reported to evaluate the performance of the predicted model.

**Results:**

In the adjusted II model**,** CCI was an independent prognostic factor for readmission within six months in patients with HF (*OR* = 1.14, 95% *CI*: 1.03–1.26, *P* = 0.011). Trend tests revealed that there was a significant linear trend for the association**.** A nonlinear association was identified between them and the inflection point of CCI was 1. Subgroup analyses and tests for interaction indicated that cystatin played an interactive role in the association. ROC analysis indicated neither CCI alone nor combinations of variables based on CCI were adequate for prediction.

**Conclusion:**

CCI was independently positively correlated with readmission within six months in patients with HF in Chinese population. However, CCI has limited value on predicting readmission within six months in patients with HF.

**Supplementary Information:**

The online version contains supplementary material available at 10.1186/s12872-023-03151-9.

## Introduction

Heart failure (HF) is one of the most life-threatening conditions in cardiovascular patients in China. A recent national epidemiologic survey in 2012–2015 revealed that the number of patients with HF has reached 89 million in China [1, 2]. Despite significant developments in cardiovascular therapy, the high rehospitalization rate has not changed significantly for the last twenty years. It is estimated that about 50% of patients are re-hospitalized within six months following a first hospitalization for HF [3].

It has become a consensus that cardiovascular and non-cardiovascular comorbidities are frequently observed in more than half of patients with HF [4], such as myocardial infarction, cerebrovascular diseases, diabetes mellitus, and so on. Also, there is evidence that comorbidities complicate the therapeutic management and contribute to poor prognosis in patients with HF [5, 6]. Consequently, the prompt recognition of associated comorbid conditions is of great significance to optimize the clinical management, the follow-up, and the treatment of patients with HF. To objectively assess comorbid status,we used the Charlson Comorbidity Index (CCI) as a surrogate marker of comorbidity[7]. Substantial researches have been performed to verify the independent correlation between CCI and short-term readmission in patients with HF in western countries[8, 9]. However, there is a scarcity of strong scientific evidence supporting the correlation in Chinese settings. The present study was therefore aimed at investigating this correlation in Chinese population. The following hypothesis was proposed in this study: Hypothesis 1. CCI was positively independently associated with readmission within six months among patients with HF in Chinese. Hypothesis 2. There may be a threshold effect or inflection point between them in Chinese.

## Methods

### Study population

**Fig. 1:**
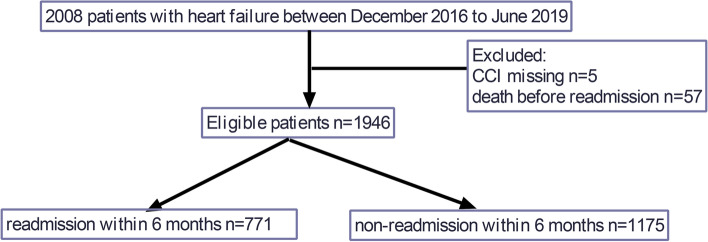
The flowchart of the participants selection

Pertinent data were obtained from an online open-source database of patients with HF in China, which is available in PhysioNet (https://physionet.org/content/heart-failure-zigong/1.2/) [10, 11]. The database consecutively and retrospectively collected electronic healthcare records of 2,008 patients with HF who had been admitted to Zigong Fourth People’s Hospital between December 2016 to June 2019. The database included all types of HF including acute heart failure (AHF), acute exacerbation of chronic heart failure (CHF), left HF, right HF, or a mixture of left and right HF. Patients with HF were identified using International Classification of Diseases (ICD)-9 code. HF was diagnosed according to the 2016 European Society of Cardiology (ESC) criteria [12]. In present study, only patients with no missing CCI were retained. As death was a competing event for readmission, we also excluded died patients before readmission. Thus, a total of 1,946 patients was included in our analysis. The flowchart of the participants selection is presented in Fig. 1. Database construction involving human participants was in accordance with the ethical standards of 1964 Declaration of Helsinki and its later amendments. Study protocol and all the methods in this study were approved by ethics committee of Zigong Fourth People’s Hospital (Approval Number: 2020–010). Ethics committee of Zigong Fourth People’s Hospital waived off the informed consent due to the retrospective design of the study [13, 14].

### Data collection

CCI was calculated by summing all comorbidity points described in the database [11]. Demographic and clinical data, including age, sex, body mass index (BMI), occupation, CCI, admission ward, admission way, discharge days (calculated by subtracting day of admission from day of discharge), body temperature, pulse, respiration, systolic blood pressure (SBP), diastolic blood pressure (DBP), New York Heart Association (NYHA) cardiac function classification, Killip grade, type of HF, type II respiratory failure and Glasgow Coma Scale (GCS) were all collected on admission. Also, a total of eight HF-related laboratory indicators on admission were included in the present study. The indicators were as follow: glomerular filtration rate (GFR), cystatin, white blood cell (WBC), hemoglobin (HGB), high sensitivity troponin T (hs-TnT), brain natriuretic peptide (BNP), high sensitivity C reactive protein (HSCRP), albumin (ALB). Left ventricular ejection fraction (LVEF) on admission was measured on echocardiography. Details on data collection and measurement are provided in the original publications [11].

### Study endpoint

The endpoint was all-cause readmission within six months calculated from the date of index hospital admission. All-cause readmission was defined as readmission for any cause after discharge from HF. Data on hospital admissions within six months was obtained at mandatory follow‐up visit or a telephone call.

### Statistical analysis

Data were analyzed using R version 4.1.0 (http://www.R-project.org. The R Foundation) All statistical inferences were made of two-sided test, and a value of P<0.05 was considered to be statistically significant. Continuous variables that approximated the normal distribution were expressed as means ± SD, while variables with a skewed distribution were expressed as median (1st quartile-3rd quartile, Q1-Q3). For categorical variables, we report frequencies and percentages. Comparisons of the baseline characteristics between no-readmission and readmission groups were examined by independent T-test for normally distributed variables, Mann Whitney U test for nonnormally distributed variables and Chi square (χ^2^) test for categorical variables. Next univariate and multivariate logistic regression analyses were performed. Based on the Strengthening the Reporting of Observational studies in Epidemiology (STROBE) guideline [15], we displayed the results of multiple models, including non-adjusted, adjusted I, adjusted II and fully-adjusted models. Non-adjusted model was not adjusted for any confounding factors. Adjusted I model was adjusted for age and sex. Adjusted II model was adjusted for covariates using change-in-estimate (CIE) and directed acyclic graph (DAG) based on age and sex [16, 17]. Fully-adjusted model was adjusted for all mentioned 25 covariates. Then patients were grouped according to category of CCI as follows: less than 2, 2, and greater than 2. We performed linear trend test by entering the median value of each category of CCI as a continuous variable in the four regression models [18]. We also explored whether there was a possible nonlinear relationship between CCI and the endpoint (the threshold effect). We applied piece-wise regression that used a separate line segment to fit each interval. Log-likelihood ratio test (LRT) comparing one-line (non-segmented) model to segmented regression model was used to determine whether threshold exists. The inflection point that connecting the segments was based on the model that gives maximum likelihood, and it was determined using two steps recursive method [19, 20]. A generalized additive model (GAM) and smooth curve fitting (restricted cubic spline curves method) were evaluated to further characterize the shape of the association between CCI and readmission. The threshold effect analysis and smooth curve fitting were adjusted for variables in adjusted II model. Moreover, Interaction and subgroup analyses were conducted according to age, sex, BMI (< 24 and ≥ 24), occupation, admission ward, admission way, discharge days (≤ 7 and > 7), body temperature (< 37.0 and ≥ 37.0), pulse (< 100 and ≥ 100), respiration (< 20 and ≥ 20), SBP (< 140 and ≥ 140), DBP (< 90 and ≥ 90), NYHA, Killip, type of heart failure, GCS (< 15 and ≥ 15), GFR (< 90 and ≥ 90), cystatin (by median), WBC (< 10 and ≥ 10), HGB (> = 120 for male and >  = 110 for female, and < 120 for male and < 110 for female), hs-TnT (by median), BNP (by median), HSCRP, ALB (< 35 and ≥ 35) and LVEF. Each stratification was adjusted for variables in adjusted II model except for the stratification factor itself and tests for interactions among subgroups were performed using LRT. There were cases with incomplete data for some covariates. Covariates with large amounts of missing data (HSCRP and LVEF) were addressed using the dummy variable, with a category for each variable used to indicate “missing” status [21]. Then we used multiple imputations (MI) based on five replications and the chained equation approach to account for missing data for cystatin, occupation, GFR, WBC, HGB, hs-TnT, BNP and ALB (The proportion of missing value was less than 20%). Then the OR, 95% CI, and P value of logistic regression of the five replications were combined according to Rubin’s rule [20]. Additionally, we explored the potential unmeasured confounding between the CCI and the endpoint using an E-value calculator (https://www.evalue-calculator.com/) [22] The E-value quantifies the magnitude of an unmeasured confounder that could negate the observed correlation between CCI and the endpoint [23]. Finally, receiver-operating characteristic (ROC) curve analysis using logistic regression was conducted, and areas under the curve (AUC), sensitivity and specificity were reported to evaluate the performance of CCI alone, CCI plus every single covariate and the combinations of variables in above-mentioned models for predicting the readmission within six months.

## Results

### Baseline characteristics

**Fig. 2:**
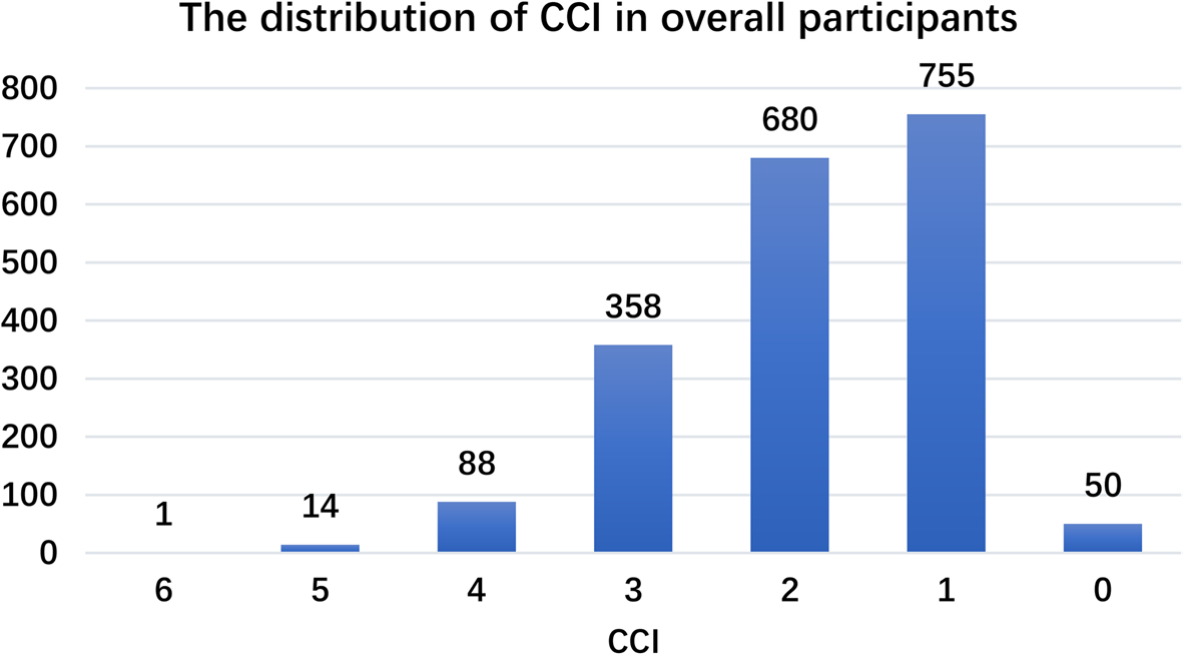
The distribution of CCI in overall participants

A total of 1,946 patients with HF were included in the study. The percentage of readmission within six months were 39.62%. The proportion of < 60 years, ≥ 60 and < 90 years, and ≥ 90 and < 110 years of patients were 8.84%, 54.06% and 37.10%, respectively. The proportion of male was 41.73%. The distribution of CCI in overall participants is shown in Fig. 2.

There were no significant differences in age, sex, BMI, admission way, body temperature, respiration, type II respiratory failure, GCS, WBC, HSCRP, ALB and LVEF between readmission and no readmission groups. Compared with no readmission group, CCI, proportion of urban resident, proportion of cardiology ward on admission, discharge day, proportion of grade IV of NYHA, proportion of grade IV of Killip, proportion of both left and right HF, cystatin, hs-TnT and BNP in readmission group were higher than no readmission group. Pulse, SBP, DBP, GFR and HGB were lower in readmission group as compared to the no readmission group. The baseline characteristics of the 1,946 included patients are shown in Table 1.

**Table 1:** Baseline characteristics of the included patients

Readmission within six months	No	Yes	*P* value
N	1175	771	
CCI	1.79 ± 0.89	1.96 ± 1.03	< 0.001
age (years)			0.469
< 60	111 (9.45%)	61 (7.91%)	
60–89	627 (53.56%)	425 (55.12%)	
90–110	437 (37.19%)	285 (36.96%)	
Sex			0.289
female	696 (59.23%)	438 (56.81%)	
male	479 (40.77%)	333 (43.19%)	
BMI (kg/m^2^)	20.83 (18.61–23.46)	20.40 (18.37–23.44)	0.097
Occupation			< 0.001
Urban resident	956 (82.06%)	663 (87.81%)	
Farmer	142 (12.19%)	48 (6.36%)	
Others	67 (5.75%)	44 (5.83%)	
Admission ward			0.006
Cardiology ward	884 (75.23%)	617 (80.03%)	
General ward	157 (13.36%)	99 (12.84%)	
Others	134 (11.40%)	55 (7.13%)	
Admission way			0.964
Emergency	559 (47.57%)	366 (47.47%)	
Non-emergency	616 (52.43%)	405 (52.53%)	
Discharge day	7.00 (6.00–10.00)	8.00 (6.00–11.00)	< 0.001
Body temperature (℃)	36.43 ± 0.44	36.39 ± 0.43	0.066
Pulse (bpm)	85.94 ± 22.16	83.96 ± 20.45	0.047
Respiration (bpm)	19.08 ± 1.79	19.06 ± 1.63	0.871
SBP (mmHg)	132.90 ± 24.42	128.49 ± 24.48	< 0.001
DBP (mmHg)	77.28 ± 14.25	75.85 ± 14.49	0.031
NYHA			< 0.001
II	240 (20.43%)	103 (13.36%)	
III	625 (53.19%)	394 (51.10%)	
IV	310 (26.38%)	274 (35.54%)	
Killip			0.015
I	324 (27.57%)	199 (25.81%)	
II	602 (51.23%)	408 (52.92%)	
III	232 (19.74%)	137 (17.77%)	
IV	17 (1.45%)	27 (3.50%)	
Type of heart failure			< 0.001
Left	336 (28.60%)	130 (16.86%)	
Right	33 (2.81%)	18 (2.33%)	
Both	806 (68.60%)	623 (80.80%)	
Type II respiratory failure			0.650
No	1110 (94.47%)	732 (94.94%)	
Yes	65 (5.53%)	39 (5.06%)	
GCS	14.90 ± 0.82	14.91 ± 0.73	0.819
GFR (ml/min)	69.85 (46.15–94.23)	57.72 (39.13–82.29)	< 0.001
Cystatin (mg/l)	1.47 (1.18–2.04)	1.64 (1.27–2.29)	< 0.001
WBC (10^9^/l)	7.34 ± 3.56	7.16 ± 3.30	0.266
HGB (g/l)	116.28 ± 23.90	113.72 ± 25.02	0.025
hs-TnT (pg/ml)	0.05 (0.02–0.11)	0.06 (0.03–0.12)	< 0.001
BNP (pg/ml)	694.89 (293.63–1632.27)	861.39 (312.25–1883.15)	0.047
HSCRP (mg/l)			0.070
< = 5	190 (16.17%)	113 (14.66%)	
> 5	383 (32.60%)	222 (28.79%)	
Missing	602 (51.23%)	436 (56.55%)	
ALB (g/l)	36.41 ± 4.97	36.85 ± 4.94	0.064
LVEF (%)			0.828
< 45	121 (10.30%)	83 (10.77%)	
> = 45	249 (21.19%)	170 (22.05%)	
Missing	805 (68.51%)	518 (67.19%)	

### Results of univariate analysis

In univariate analysis, CCI, occupation, admission ward, discharge day, pulse, SBP, DBP, NYHA, Killip, type of HF,GFR, cystatin and HGB were associated with readmission within six months (P < 0.05). The results of the univariate analyses are presented in Supplementary Table S1.

### Results of multivariate analysis and trend test

In non-adjusted model, CCI was positively correlated with readmission within six months (*OR* = 1.20, 95% *CI*: 1.09–1.32, *P* < 0.001). In the adjusted I and II models, ORs of the positive association were listed as follows: *OR* = 1.19, 95% *CI*: 1.08–1.31, *P* < 0.001 and *OR* = 1.14, 95% *CI*: 1.03–1.26, *P* = 0.011. In fully-adjusted model, CCI was also positively related with the endpoint (*OR* = 1.18, 95% *CI*: 1.05–1.32, *P* = 0.006). Trend tests revealed that there was a linear trend for the association between CCI and readmission and the linear trend tests were significant in the four models (*P* for trend < 0.05). *OR* for the difference between CCI <  = 1 and CCI = 2 group appeared quite different from that between CCI = 2 and CCI > 2 group. This suggested a possible threshold effect in this relationship which becomes more noticeable when the threshold is exceeded. The results of multivariate analysis and trend test are shown in Table 2

**Table 2:** Results of the multivariate analysis and trend test between CCI and readmission

model	non-adjusted	adjust I	adjust II	fully-adjusted
N	1946	1946	1867	1686
CCI	1.20 (1.09, 1.32) < 0.001	1.19 (1.08, 1.31) < 0.001	1.14 (1.03, 1.26) 0.011	1.18 (1.05, 1.32) 0.006
CCI groups				
< = 1	*Ref*	*Ref*	*Ref*	*Ref*
2	1.08 (0.88, 1.34) 0.460	1.08 (0.87, 1.33) 0.501	1.05 (0.84, 1.31) 0.694	1.02 (0.80, 1.30) 0.852
> 2	1.53 (1.21, 1.93) < 0.001	1.51 (1.19, 1.91) 0.001	1.36 (1.05, 1.75) 0.018	1.44 (1.09, 1.92) 0.011
*P* for trend	0.001	0.001	0.026	0.019

The values of the table are *OR*, 95%*CI* and *P* value for the association between CI and readmission

Non-adjusted model adjusted for: None

Adjusted I model adjusted for: age, sex

Adjusted II model adjusted for: age, sex, type of HF, NYHA, GFR, cystatin, discharge day

Fully adjusted model adjusted for: age, sex, BMI, occupation, admission ward, admission way, discharge day, body temperature, pulse, respiration, SBP, DBP, NYHA, Killip, type of heart failure, type II respiratory failure, GCS, GFR, cystatin, WBC, HGB, hs-TnT, BNP, HSCRP, ALB and LVEF

### Non-linearity correlation between CCI and readmission with six months

**Fig. 3:**
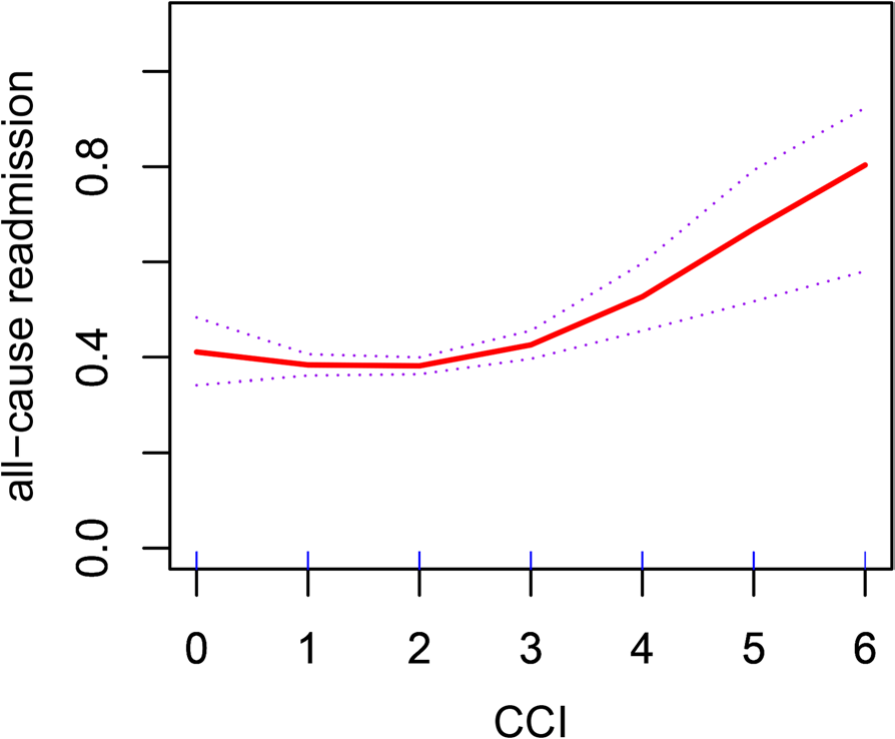
The smooth curve fitting between CCI and readmission

Adjusted smooth curve fitting showed that the relationship between CCI and readmission was nonlinear (Fig. 3). The threshold effect analysis revealed the inflection point of CCI was 1 after adjusting covariates in adjusted II model (*P* for LRT = 0.007 < 0.05). The correlation was significantly negative before the inflection (*OR* = 0.51, 95%*CI*: 0.28–0.92,* P* = 0.026) while the correlation became significantly positive after the inflection (*OR* = 1.20, 95%*CI*: 1.08–1.34,* P* = 0.007). As a result, we concluded that the correlation between CCI and readmission was nonlinear.

### Subgroup analysis and tests for interaction

The subgroup analyses and tests for interaction of the correlations between CCI and the endpoint are presented in Supplementary Table S2. The positive correlations between CCI and the endpoint were stable in nearly all subgroups (*OR* > 1). The interaction analysis revealed that cystatin played a significant interactive role in the association between CCI and readmission (*P* for interaction = 0.042). The patients with cystatin < 1.55 mg/l had a much worse prognosis (*OR* = 1.31, 95% CI: 1.11–1.53) than those with cystatin ≥ 1.55 mg/l (*OR* = 1.04, 95% CI: 0.91–1.18). No other significant interaction was observed in other subgroup analyses.

### Multiple imputations of missing values

We found that some variables for HSCRP and LVEF, cystatin, occupation, GFR, WBC, HGB, hs-TnT, BNP and ALB were missing in raw data and the numbers of missing were 1038, 1323, 40, 26, 62, 26, 27, 77, 34 and 96, respectively. Dummy variable and MI were used to handle missing values. The results of the MI indicated that there was only a slight difference in *OR*s for four logistic models between raw data and combined five imputed replications. In other words, we concluded that the data for cystatin, occupation, GFR, WBC, HGB, hs-TnT, BNP and ALB appeared to be missing at random, which would not significantly alter the results of raw data. A summary of imputed data compared with the initial incomplete data is illustrated in Supplementary Table S3.

### The ROC analysis for CCI predicting readmission

The AUC for CCI alone predicting readmission with six months was only 54.18% (95%*CI*: 51.67–56.68%). The specificity and sensitivity were 79.15% and 28.02%, respectively. Besides, neither CCI plus every single covariate nor the combinations of variables in logistic models displayed good performance for predicting readmission (AUCs < 70.00%). The results of ROC analysis are listed in Supplementary Table S4.

## Discussion

Significant comorbidities are common in patients with HF and higher comorbidity burden was associated with greater possibility of HF hospitalization, as we have experienced in clinical practice. CCI is the most widely known standardized comorbidity score. Consequently, present study explored the correlation between CCI and readmission with six months in HF for this purpose.

Previous studies have mainly confirmed the positive correlation between CCI and readmission in western countries. A national retrospective cohort study in the Netherlands reported that a higher CCI increased the risk of early readmission in patients ≥ 70 years with HF within 7, 30 and 42 days [8]. In a single-centered retrospective cohort study in the U.S., Daniel Keyes et.al confirmed that CCI were significantly lower for geriatric patients with HF in the > 30-day/non-readmitted subgroup compared to earlier readmission patients [9]. Our study further provided complementary evidence and corroborated the correlation between CCI and readmission in Chinese. Thus, current evidence points toward the existence of the association, no matter in western countries or in Chinese.

In our study, the patients with higher CCI had a greater likelihood of readmission with six months than those with a lower CCI (adjust II model: *OR* = 1.14, 95% *CI*: 1.03–1.26, *P* = 0.011, *P* for trend = 0.026). This is consistent with the literature reported previously. We further found a curvilinear correlation between them (LRT P > 0.05) and the inflection of CCI was 1. This is the first study to explore the threshold effect between them in patients with HF. Based on the previous results, we think that CCI > 1 appears to have a more strongly connection to readmission. Also, we first found cystatin played a significant interactive role in the association between CCI and readmission and it was suggested that the patients with cystatin < 1.55 mg/l showed higher readmission rate. However, there are no reports on this problem and it is required to investigate it further. Although our study demonstrates a causal relationship between CCI and readmission, it needs to be noted that CCI alone or the combinations of variables based on CCI was inadequate for predicting readmission. According to a previous meta-analysis, most AUCs for readmission predictive models in external validation cohort in patients with HF was below 0.75 and the most common predictors in the model were BNP/pro-BNP and renal insufficiency [24]. These may suggest that predicting readmission is a difficult subject in the field of HF because of the heterogeneous and complexity nature of HF. It also reminds us that BNP/pro-BNP and renal insufficiency could be better predictors compared with CCI.

We recognized some limitations of our study as well. First, this study represents a retrospective analysis, with inherent biases associated with this study design. Second, all subjects included were Chinses, and therefore our findings may not be extrapolated to other populations. Third, our cohort inevitably included a proportion of missing covariate data but it was addressed by dummy variables and multiple imputations in this study. Fourth, we did not perform survival analysis due to no follow-up information recorded in raw data for overwhelming majority of the participants. Fifth, as with any observational study, there is an unavoidable potential for residual confounding such as medication. However, based on E-value computations, changes to our results from unmeasured confounding would be unlikely (E-value = 1.34). E-values estimated in our study suggest that a confounder must have relative strong associations with both CCI and readmission within six months simultaneously (relative risk ≥ 1.34) to completely dilute the observed association [25]. Sixth, the original study enrolled AHF and CHF but failed to distinguish them from each other. Since AHF and CHF show dramatic differences in pathogenesis and prognosis, it might be possible that the strength of association between CCI and readmission in AHF and CHF.

## Conclusion

In Chinese population, CCI was independently positively correlated with readmission within six months in patients with HF. However, CCI has limited value on predicting readmission within six months in patients with HF. As this study has several limitations, the principal conclusions of this paper need to be taken with caution.

## Supplementary Information

**Additional file 1: Table S1. **Results of univariate analysis. **Table S2.** Subgroup analysis and tests for interaction. **Table S3.** Results of multivariate analysis based on five multiple imputations. **Table S4.** The ROC analysis for CCI predicting readmission.  

## Data Availability

The data used in this study are available online in PhysioNet (https://physionet.org/content/heart-failure-zigong/1.2/).

## Abbreviations

HF: Heart failure
CCI: Charlson Comorbidity Index
AHF: Acute heart failure
CHF: Chronic heart failure
ICD: International Classification of Diseases
ESC: European Society of Cardiology
BMI: Body mass index
SBP: Systolic blood pressure
DBP: Diastolic blood pressure
NYHA: New York Heart Association
GCS: Glasgow Coma Scale
GFR: Glomerular filtration rate
WBC: White blood cell
HGB: Hemoglobin
hs-TnT: High sensitivity troponin
BNP: Brain natriuretic peptide
HSCRP: High sensitivity C reactive protein
ALB: Albumin
LVEF: Left ventricular ejection fractions
CI: Confidence Interval
Q1: 1St quartile
Q3: 3Rd quartile
χ 2: Chi-square
STROBE: Strengthening the Reporting of Observational studies in Epidemiology
CIE: Change-in-estimate
DAG: Directed acyclic graph
LRT: Likelihood ratio test
GAM: Generalized additive model
MI: Multiple imputations
ROC: Receiver-operating characteristic
AUC: Areas under the curve
